# A novel pinless augmented reality-based navigation system for total hip arthroplasty in the supine position: a comparative study of acetabular cup angle accuracy

**DOI:** 10.1007/s00402-026-06324-1

**Published:** 2026-05-15

**Authors:** Hiroaki Kurishima, Hiroyuki Ogawa, Norikazu Yamada, Atsushi Noro, Yasutake Tomata, Sachiyuki Tsukada

**Affiliations:** 1 Department of Orthopaedic Surgery, Japanese Red Cross Sendai Hospital, Sendai, Japan; 2 Department of Orthopaedic Surgery, Tohoku University Graduate School of Medicine, Sendai, Japan; 3 Department of Orthopaedic Surgery, Hokusuikai Kinen Hospital, Mito, Japan; 4 Faculty of Health and Social Service, Kanagawa University of Human Services, Yokosuka, Japan

**Keywords:** Computer-assisted surgery, Portable navigation system, Anterolateral-supine approach, Smartphone

## Abstract

**Introduction:**

We developed a novel pinless augmented reality (AR)-based portable navigation system for total hip arthroplasty (THA) in the supine position to eliminate pin-site complications and intraoperative navigation abandonment, and to reduce registration time. This study compared the measurement accuracy of this new pinless portable navigation system with that of a conventional AR-based portable navigation system requiring pin insertion.

**Materials and methods:**

This retrospective cohort study compared primary unilateral THAs performed using the novel pinless AR-based navigation system and a conventional AR-based navigation system. The primary outcome was the absolute difference between the acetabular cup angles displayed on the navigation screen and those measured on postoperative computed tomography. Secondary outcomes included target achievement rates within 5° and 10°. Pin-site complications, intraoperative navigation abandonment, and dislocations were also recorded.

**Results:**

A total of 228 hips were analyzed (Pinless: *n* = 115; Conventional: *n* = 113). While there was no significant difference in the median absolute difference for inclination (2.5° vs 2.4°; *p* = 0.366), the median absolute difference for anteversion was significantly larger in the pinless group than in the conventional group (2.9° vs 1.9°; *p* = 0.011). There were no significant differences in the percentages within 5° (80.0% vs 77.9%; *p* = 0.747) and 10° (99.1% vs 97.3%; *p* = 0.367). No pin-site complications, intraoperative navigation abandonment, or dislocations occurred in either group.

**Conclusions:**

Despite a small statistical difference in the absolute anteversion error, the pinless AR-based navigation system demonstrated acceptable measurement accuracy and comparable target achievement rates relative to the conventional system requiring pin insertion in this retrospective cohort. While the new pinless AR-based navigation system eliminates the risk of pin-site complications and intraoperative navigation abandonment, and offers instantaneous registration, further prospective studies are warranted to confirm these findings.

## Introduction

In total hip arthroplasty (THA), various computer-assisted systems such as robot-assisted systems, computed tomography (CT)-based navigation, and several types of portable navigation have been developed to improve the accuracy of acetabular cup placement and to reduce the risk of dislocation and polyethylene wear [1]. Robot-assisted systems and CT-based navigation have been reported to provide greater accuracy in both the angle and position of the acetabular cup compared with portable navigation [2]. However, not all medical institutions can implement these systems because of the high acquisition and maintenance costs [3].

Many existing robot-assisted systems [4, 5], CT-based navigation systems [6], and portable navigation systems [7–11] require the insertion of pins to fix markers to the pelvis and require registration of the pelvic position. However, pin-site complications [12] and intraoperative navigation abandonment due to pin loosening [13] have been reported. Furthermore, the insertion of pins into the pelvis can prolong the operation time, which may increase the overall complication rate [14]. Although a noninvasive robot-assisted system [15] and a portable navigation system [16] for the supine position have been developed, the process of registering pelvic anatomical landmarks remains time-consuming [4, 5, 7–11, 15, 16].

**Fig. 1:**
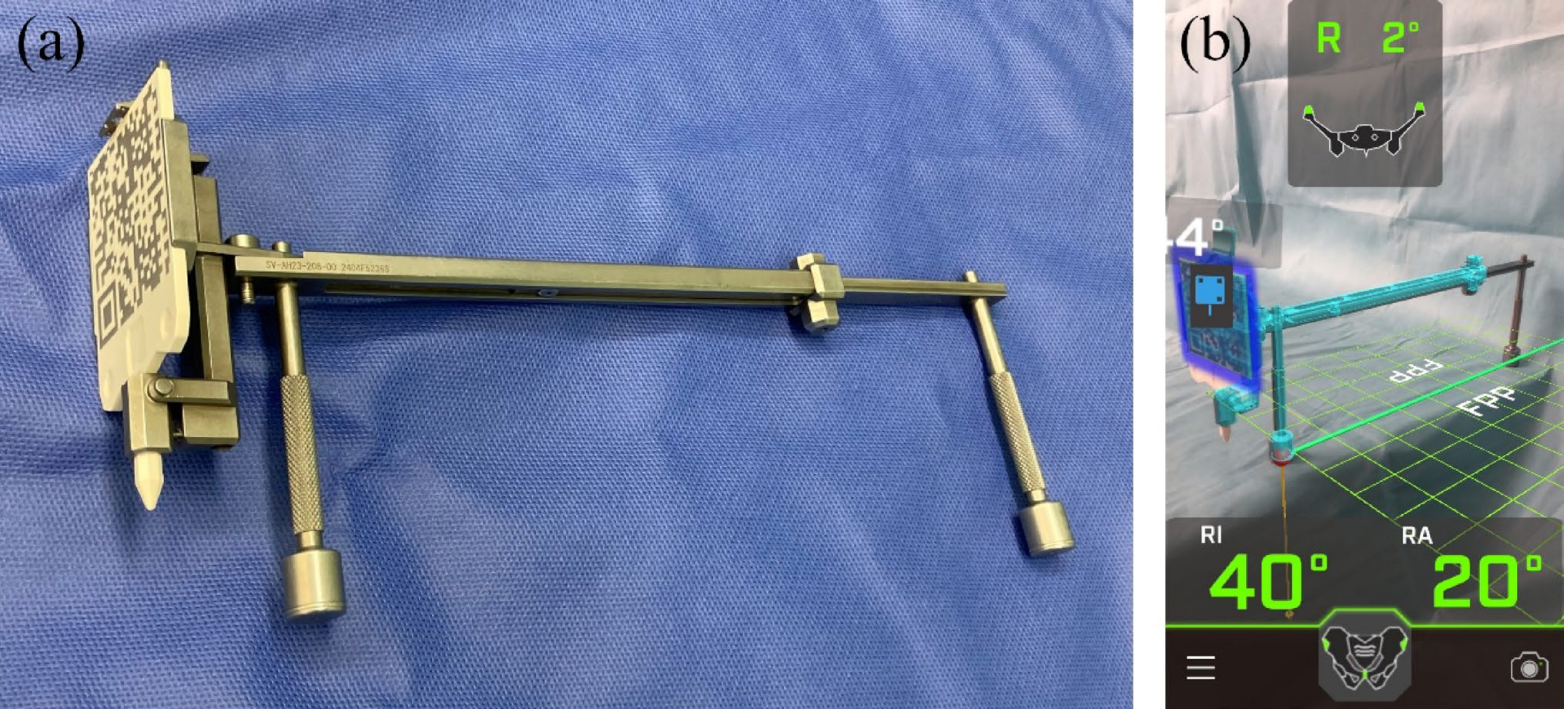
The novel pinless AR-based portable navigation system. **a** The specialized device equipped with a marker. **b** The navigation screen display. The lateral pelvic tilt angle is shown at the top, the FPP is visualized in the center, and the cup angles (RI, radiographic inclination; RA, radiographic anteversion) relative to the FPP are displayed at the bottom. *AR* augmented reality, *FPP* functional pelvic plane

To address these issues, we developed a novel pinless augmented reality (AR)-based portable navigation system for THA in the supine position. This new system features a specialized device equipped with a marker (Fig. 1a). Once the smartphone application recognizes this marker, the functional pelvic plane (FPP) is calculated based on the orientation of the bar and the direction of gravity detected by the smartphone’s internal gyroscope, and the cup angle relative to the FPP is displayed on the screen (Fig. 1b). Registration is performed nearly instantaneously by simply positioning this device on the bilateral anterior superior iliac spines (ASIS), allowing for simultaneous measurement of the cup angle. While this new pinless AR-based navigation system can algorithmically track axial and coronal pelvic rotation, it cannot detect intraoperative changes in sagittal pelvic tilt. Due to this structural limitation, although the high accuracy of AR-based portable navigation has been reported [11, 17, 18], this new AR-based navigation system may potentially reduce the accuracy of the acetabular cup angle.

This study aimed to compare the accuracy of the acetabular cup angle in THA between the novel pinless AR-based portable navigation system and a conventional AR-based navigation system. Our hypothesis was that there would be no significant difference in cup angle accuracy between the two navigation systems, leading to equivalent target achievement rates.

## Materials and methods

### Study design and setting

This sequential, nonrandomized, and retrospective cohort study was approved by our Institutional Review Board (approval number: 2023-01) and analyzed data collected between December 2023 and November 2024 at a single high-volume institution (Japanese Red Cross Sendai Hospital).

### Study participants

We reviewed consecutive patients who underwent primary unilateral THA performed by three experienced hip surgeons (NY, AN, and HK) using the anterolateral approach in the supine position with one of the two specified AR-based navigation systems. Patients were divided into two groups based on the time period. All three surgeons utilized the conventional AR-based portable navigation system until June 2024 (Conventional group). Subsequently, all three surgeons transitioned simultaneously to the novel pinless AR-based portable navigation system starting in July 2024 (Pinless group).

All surgeons had performed over 150 THAs using portable navigation before the study period. To ensure familiarity with the new device, the novel pinless system was used concurrently with the conventional system during the final month of the conventional group period. In these cases, the conventional system remained the primary guide for cup insertion.

Patients with a history of pelvic osteotomy, intraoperative cup translation, postoperative fracture around the acetabular cup, or incomplete data—such as navigation data, postoperative CT, or standing spine radiography—were excluded. Additionally, cases in which the target angle was adjusted because of patient-specific spinopelvic parameters (posterior pelvic tilt change > 10° from supine to standing) or excessive femoral anteversion (> 45°) were excluded from the analysis.

### Surgical technique

The surgical technique has been described previously [19, 20]. Fluoroscopy was used for intraoperative verification of component position and size, except for acetabular cup angles. A single cementless acetabular cup (G7; Zimmer Biomet, Warsaw, IN) was implanted in all cases. Unless adjustments were required (which was an exclusion criterion), the target angle was 40° for inclination and 20° for anteversion, relative to the FPP. Postoperatively, all patients were permitted immediate full weight bearing, as tolerated, without additional activity restrictions.

### Two types of augmented reality-based portable navigation systems

Two types of AR-based portable navigation systems were used. The first was the conventional system (AR-Hip system; Zimmer Biomet Japan, Tokyo, Japan), which utilized pins inserted into the iliac crest. The AR-Hip system has been described previously [2, 11]. The second was a novel pinless system (Pinless AR-Hip system; Zimmer Biomet Japan) that employs a specialized device. Both navigation systems share the same underlying smartphone-based AR technology; the FPP is calculated by registering the bilateral ASIS with markers and sensing the direction of gravity via a gyro sensor, and the cup placement angle relative to the FPP is displayed on the screen [17].

**Fig. 2:**
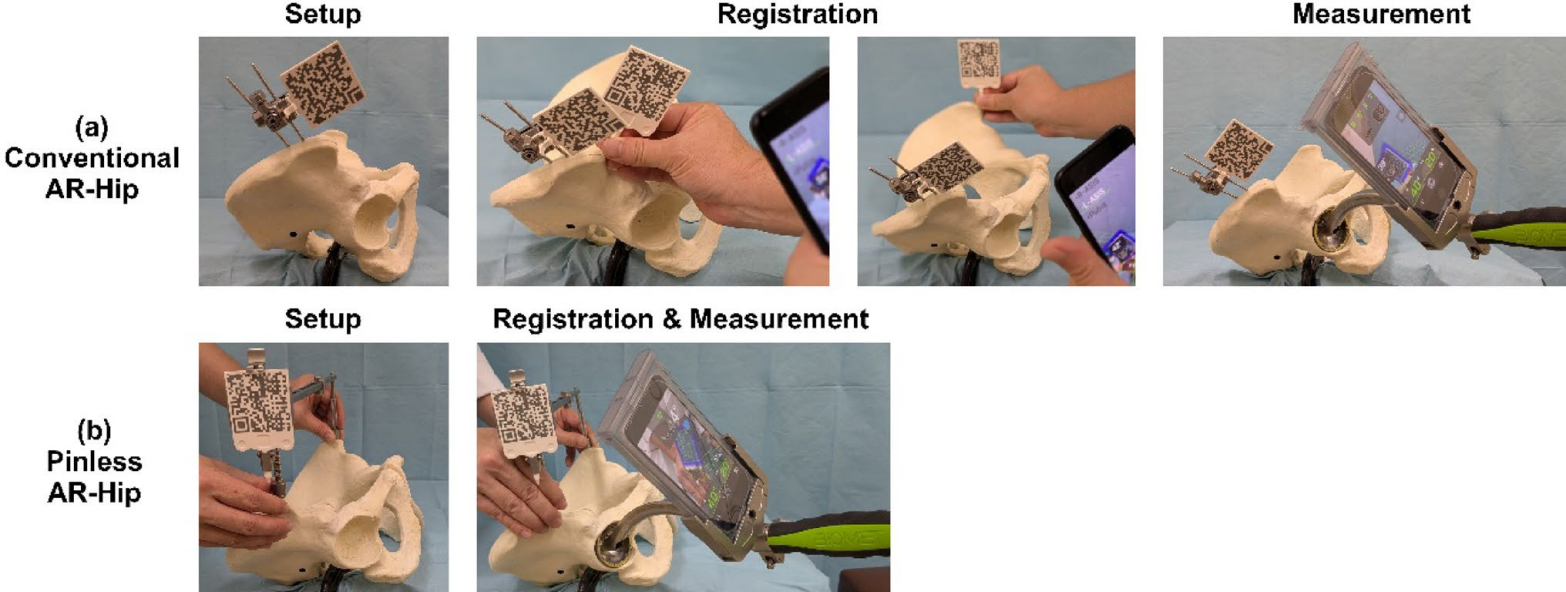
Comparison of the registration methods. **a** The conventional AR-Hip system requires pin insertion into the iliac crest for marker fixation. This method involves sequential registration of the bilateral ASIS, followed by the measurement of the acetabular cup angle. **b** The Pinless AR-Hip system requires only the positioning of the specialized device on the bilateral ASIS. Registration and measurement of the acetabular cup angle are performed simultaneously. *AR* augmented reality, *ASIS* anterior superior iliac spine

However, the FPP registration methods differ between the two systems. The conventional AR-Hip system requires two markers and pins. The first marker is fixed to the iliac crest with pins, and the second marker is used to sequentially register the bilateral ASIS to establish the FPP (Fig. 2a). During acetabular cup placement, the smartphone is attached to the cup impactor, and the acetabular cup angle is calculated relative to the first marker (Fig. 2a). In contrast, the Pinless AR-Hip system utilizes a specialized device with a single marker, allowing for simultaneous FPP registration and cup angle measurement. The FPP is established simply by positioning the device on the bilateral ASIS, and the smartphone attached to the cup impactor concurrently displays the cup angle (Fig. 2b). While the conventional AR-Hip system requires time for pin insertion, marker base attachment, and two-step ASIS registration, the Pinless AR-Hip system establishes the FPP almost instantaneously. In this study, registration for the conventional system was performed immediately before surgical incision; therefore, this registration time was not included in the operative time.

These registration methods yielded several distinct features. In the conventional AR-Hip system, once registration in the supine position is complete, the pelvic motion can be tracked continuously due to the marker fixed to the pelvis. Therefore, the conventional AR-Hip system was compatible with both supine and lateral decubitus positions and does not require the removal of the retractor or pillows. However, with the Pinless AR-Hip system, registration and measurement could be performed only with the patient in the supine position because these processes occur simultaneously. In addition, the Pinless AR-Hip system could track axial and coronal pelvic rotation but could not detect intraoperative changes in sagittal tilt. Algorithmically, the system assumes the sagittal tilt always remains in an ideal neutral position, regardless of actual intraoperative pelvic movement. To minimize error caused by sagittal pelvic tilt, it is essential to remove any retractors and pillows under the leg to reproduce the neutral FPP during measurement.

### Data collection/Measurements

Postoperative CT scans were obtained 1–6 months after surgery. The primary author (HK), who was blinded to patient characteristics and the navigation system used, measured the radiographic inclination and anteversion of the acetabular cup relative to the FPP. For these measurement, a three-dimensional template of the acetabular cup was manually applied using three-dimensional software (ZedHip; LEXI Co., Tokyo, Japan) [21]. This measurement method has previously been reported to have excellent reliability (intra- and interobserver intraclass correlation coefficients ≥ 0.97) [21]. The FPP was defined by the bilateral ASIS and the CT table plane [22].

### Outcomes

Our primary outcomes were the absolute and signed differences between the acetabular cup angles displayed on the navigation screen and those measured on postoperative CT. The absolute difference was used to assess accuracy, whereas the signed difference was used to assess systematic bias. A positive signed difference indicated that the angle displayed on the navigation system was larger than that measured on CT.

The secondary outcomes included the final acetabular cup angles, absolute differences from the target, and target achievement rates (percentage of acetabular cups placed within 5° and 10° of the target angle). Finally, safety outcomes included pin-site complications, intraoperative navigation abandonment, and dislocations during the study period.

### Data analyses

The normality of continuous variables was assessed using the Kolmogorov–Smirnov test. Data are presented as medians with interquartile ranges for non-normally distributed variables, or as means ± standard deviations for normally distributed variables. Between-group comparisons for continuous variables were performed using the Mann–Whitney *U* test (non-normal distribution) or the Student’s *t*-test (normal distribution), as appropriate. Fisher’s exact or *chi*-squared test were used for the analysis of categorical variables. Statistical significance was set at *p* < 0.05. All analyses were conducted using EZR version 1.65 [23].

## Results

**Fig. 3:**
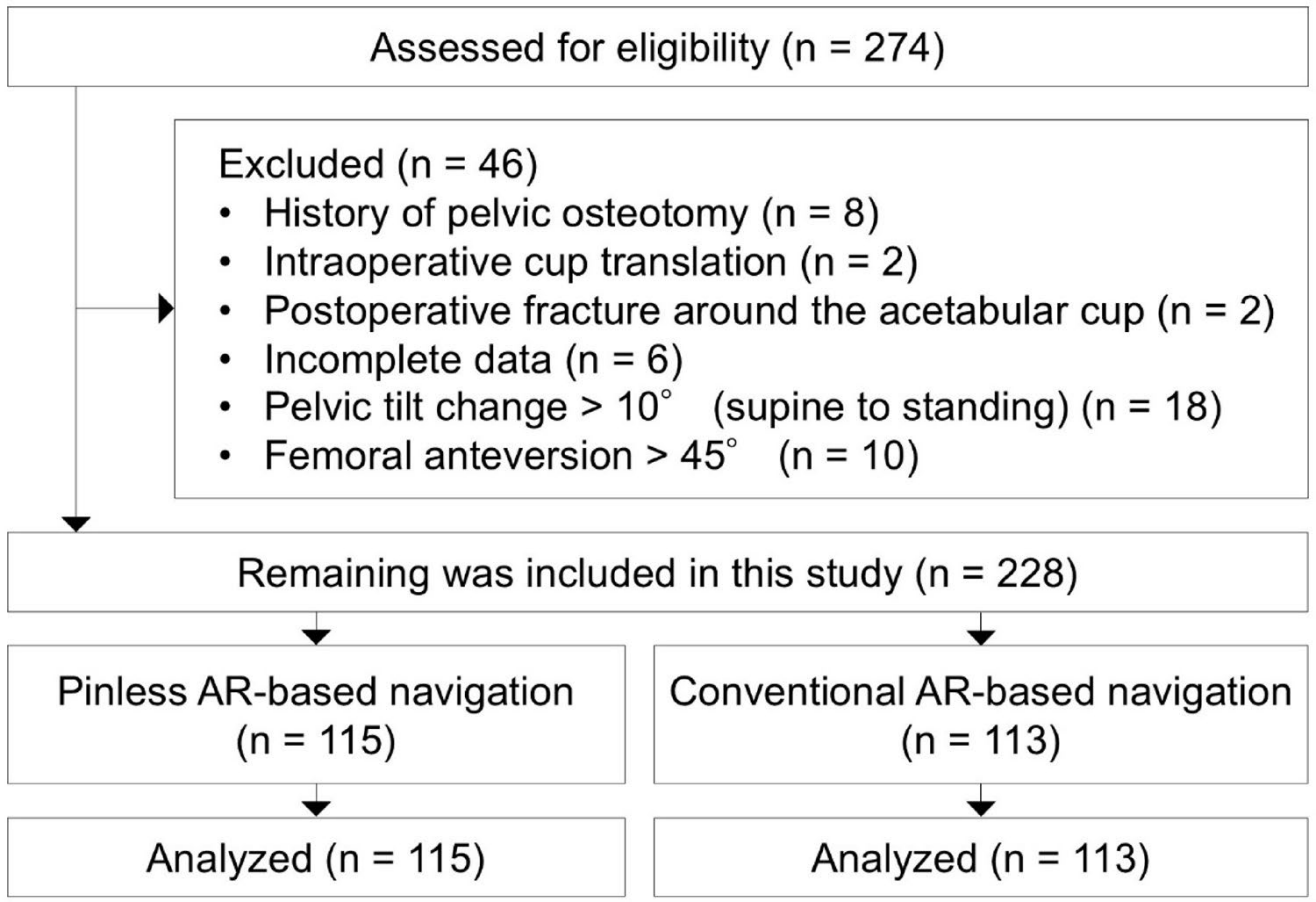
Flow diagram illustrating the patient selection process

From a total of 274 potentially eligible hips, 46 hips were excluded based on the criteria. Consequently, a final cohort of 228 hips from 219 patients was included in the analysis. The cohort consisted of the pinless group (*n* = 115) and the conventional group (*n* = 113) (Fig. 3). With the current sample size, the study achieved a statistical power exceeding 80%, based on an assumed mean difference of 0.75° and a standard deviation of 2.0°. There were no significant differences in baseline demographic characteristics between the two groups, with the exception of the follow-up duration (Table 1).

**Table 1:** Patient Demographic and Baseline Clinical Characteristics

Characteristic	Pinless AR-based navigation (*n* = 115)	Conventional AR-based navigation (*n* = 113)	*P* value
Age (years)	69 ± 8	67 ± 9	0.195^a^
Sex (women/men)	97/18	98/15	0.707^b^
Height (cm)	155.3 ± 7.6	155.4 ± 7.6	0.922^a^
Weight (kg)	58.0 ± 10.8	59.1 ± 12.1	0.456^a^
Body mass index (kg/m^2^)	24.0 ± 3.7	24.4 ± 4.7	0.404^a^
Diagnosis (OA/ONFH/RA/fracture)	110/3/0/2	110/1/2/0	0.246^b^
Surgeon (YN/NA/HK)	28/61/26	38/49/26	0.255^b^
Duration of surgery (min)	66.8 ± 12.5	69.4 ± 17.3	0.207^a^
Intraoperative blood loss (ml)	344.6 ± 195.2	326.9 ± 157.1	0.454^a^
Duration of follow-up (months)	11.8 ± 1.4	12.5 ± 2.0	**0.002** ^**a**^

Results are expressed as means ± standard deviations, unless otherwise indicated

*AR* augmented reality, *OA* osteoarthritis, *ONFH* osteonecrosis of the femoral head, *RA* rheumatoid arthritis

^a^*P* values were derived from Student’s *t*-test

^b^*P* values were derived from Fisher’s exact test or *chi*-squared test

Boldface indicates statistical significance (*p* < 0.05)

Assessment of normality revealed that the absolute differences between the navigation and CT measurements did not follow a normal distribution (inclination, *p* = 0.028; anteversion, *p* = 0.003), whereas the corresponding signed differences were normally distributed (inclination, *p* = 0.973; anteversion, *p* = 0.574). Similarly, the absolute differences from the target angles also did not follow a normal distribution (inclination, *p* = 0.003; anteversion, *p* = 0.002).

Table 2 presents the absolute differences between the acetabular cup angles displayed on the navigation screen and those measured on CT. While there was no significant difference in the median absolute difference for inclination (2.5° vs 2.4°; *p* = 0.366), the median absolute difference for anteversion in the pinless group was significantly larger than that in the conventional group (2.9° vs 1.9°; *p* = 0.011).

**Table 2:** The absolute differences between acetabular cup inclination and anteversion angles displayed on the navigation screen and those measured on postoperative computed tomography

	Pinless AR-based navigation (*n* = 115)	Conventional AR-based navigation (*n* = 113)	*P* value
Inclination (°)	2.5 (1.4 to 4.5)	2.4 (1.1 to 3.9)	0.366
Anteversion (°)	2.9 (1.3 to 4.4)	1.9 (1.0 to 3.6)	**0.011**

Results are expressed as medians (interquartile range)

*AR* augmented reality

*P* values were derived from Mann–Whitney *U* test

Boldface indicates statistical significance (*p* < 0.05)

**Fig. 4:**
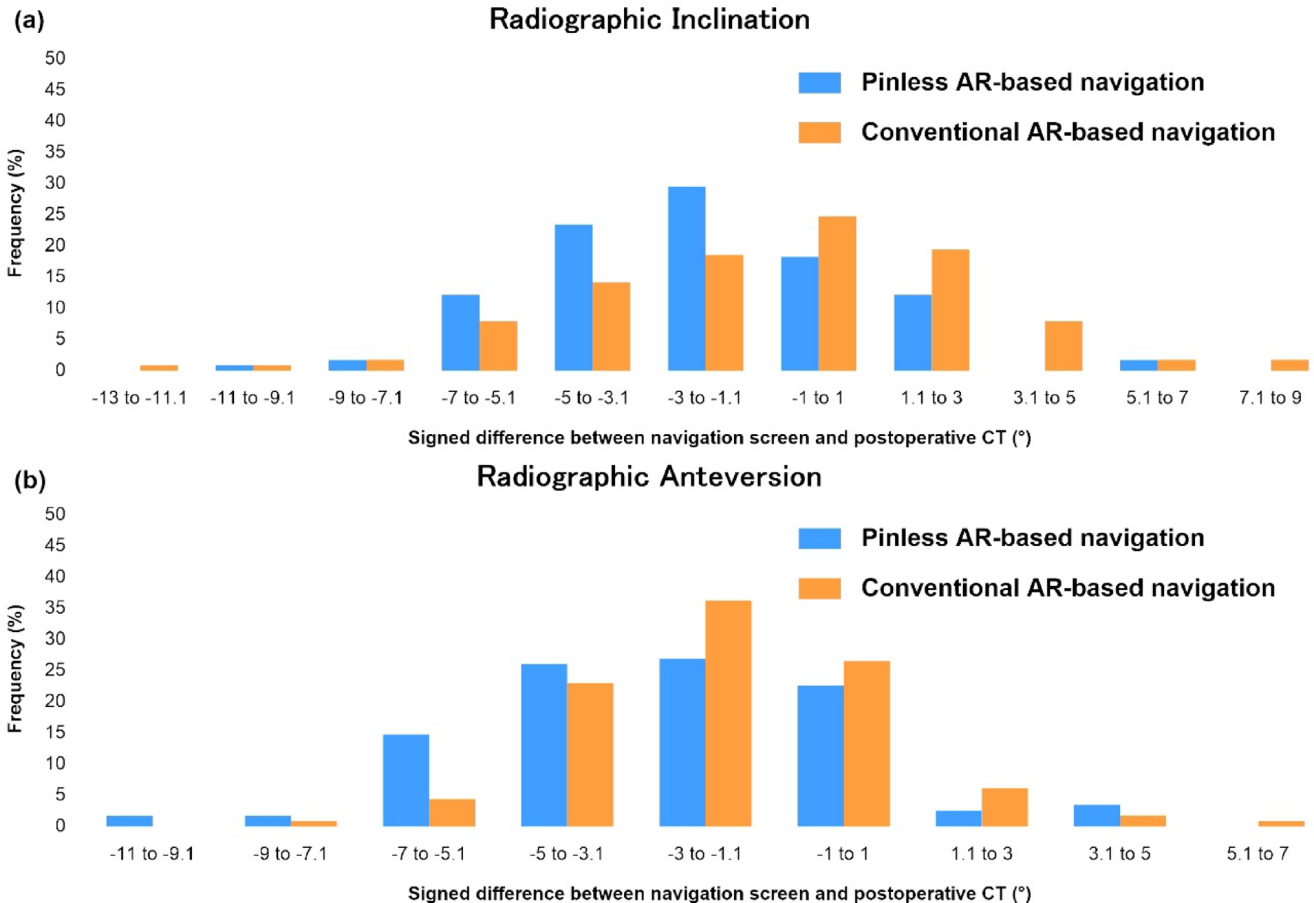
Distribution of signed differences between the angles displayed on the navigation screen and those measured on postoperative CT (defined as Navigation angle − CT angle). **a** Radiographic inclination. **b** Radiographic anteversion. *CT* computed tomography

The mean signed difference revealed a significantly different systematic bias for both inclination (-2.2° vs -0.8°; *p* < 0.001) and anteversion (-2.5° vs -1.7°; *p* = 0.017) (Table 3). Figure 4 illustrates the distribution of the signed differences. The negative mean values of the signed differences indicate a general tendency for the actual cup angles measured on postoperative CT to be larger than the displayed navigation values. As shown in the distribution, this tendency—where the actual CT angles exceed the navigation display—was significantly more pronounced in the pinless group than in the conventional group.

**Table 3:** The signed differences between acetabular cup inclination and anteversion angles displayed on the navigation screen and those measured on postoperative computed tomography

	Pinless AR-based navigation (*n* = 115)	Conventional AR-based navigation (*n* = 113)	Mean difference (95% confidence interval)	*P* value
Inclination (°)	-2.2 ± 2.8 (-9.3 to 5.8)	-0.8 ± 3.5 (-12.7 to 7.8)	-1.4 (-2.3 to -0.6)	** < 0.001**
Anteversion (°)	-2.5 ± 2.8 (-10.3 to 5.0)	-1.7 ± 2.3 (-7.8 to 6.0)	-0.8 (-1.5 to -0.2)	**0.017**

Results are expressed as means ± standard deviations (range), unless otherwise indicated

*AR* augmented reality

*P* values were derived from Student’s *t*-test

Boldface indicates statistical significance (*p* < 0.05)

**Fig. 5:**
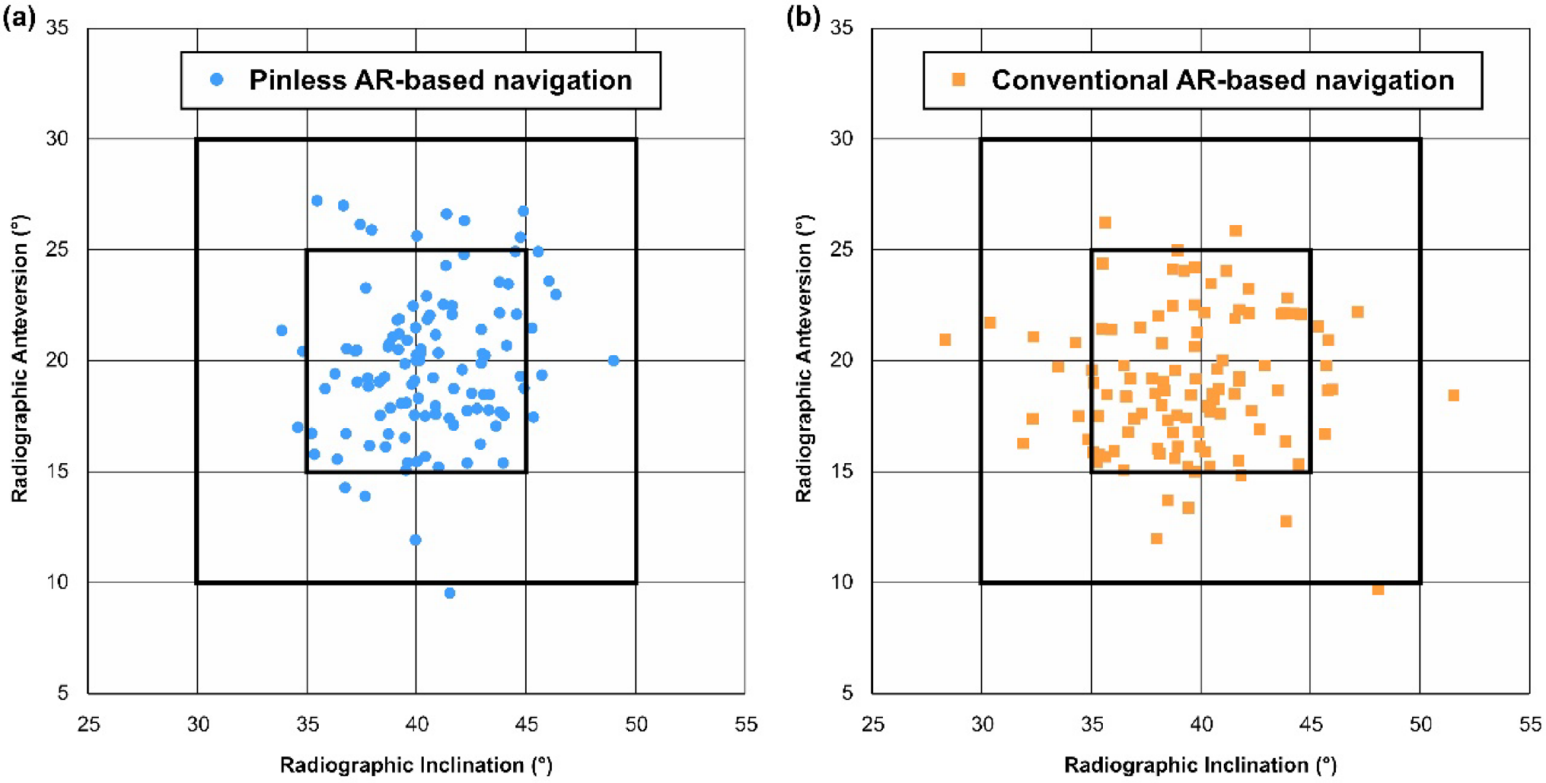
Scatter plots of postoperative acetabular cup angles measured on CT. **a** The pinless AR-based navigation group. **b** The conventional AR-based navigation group. *CT* computed tomography, *AR* augmented reality

Postoperative CT measurements of acetabular cup angles and statistical comparisons are summarized in Table 4. The mean angles were comparable between the groups. There were no significant differences between the groups regarding the median absolute differences from the target angles for both inclination and anteversion (*p* = 0.122 and *p* = 0.385, respectively). Figure 5 shows a scatter plot of the individual acetabular cup angles measured on postoperative CT. There were no significant differences in the target achievement rates within 5° (80.0% [92 of 115] vs 77.9% [88 of 113]; *p* = 0.747) and 10° (99.1% [114 of 115] vs 97.3% [110 of 113]; *p* = 0.367).

**Table 4:** Comparison of postoperative acetabular cup angles and absolute differences from the target (Target: inclination, 40°; anteversion, 20°)

	Pinless AR-based navigation (*n* = 115)	Conventional AR-based navigation (*n* = 113)	*P* value
*Postoperative acetabular cup angle measured on CT*			
Inclination (°)	40.6 ± 2.9 (33.9 to 49.0)	39.4 ± 3.8 (28.3 to 51.5)	
Anteversion (°)	19.8 ± 3.3 (9.3 to 27.2)	18.9 ± 3.0 (9.7 to 26.2)	
*Absolute difference from the target angle*			
Inclination (°)	2.1 (0.8 to 3.6)	2.0 (1.0 to 4.4)	0.122
Anteversion (°)	2.1 (1.0 to 3.7)	2.2 (1.3 to 4.0)	0.385

Postoperative acetabular cup angles measured on CT are expressed as means ± standard deviations (range), and absolute differences from the target angle are expressed as medians (interquartile range)

*AR* augmented reality, *CT* computed tomography

*P* values were derived from Mann–Whitney *U* test

There were no cases of pin-site complications or intraoperative navigation abandonment in the conventional group, and no dislocations occurred in either group.

## Discussion

Although computer-assisted navigation can improve the accuracy of component placement in THA, many conventional systems require pin insertion, which carries the risk of pin-site complications and intraoperative navigation abandonment, and increases operative time. Our novel pinless and simple AR-based navigation system eliminates these drawbacks; however, it is essential to confirm its accuracy. In this study, the novel pinless AR-based navigation system demonstrated comparable accuracy in the inclination angle, while showing a statistically larger absolute difference in the anteversion angle compared to the conventional system requiring pin insertion for THA in the supine position. We acknowledge that this larger anteversion error likely reflects a fundamental structural limitation of the pinless system: the inability to detect intraoperative changes in sagittal pelvic tilt. Nevertheless, the mean difference was less than 1 degree, and the pinless AR-based navigation demonstrated target achievement rates comparable to the conventional system. Given the practical advantages, this system represents a valuable option for THA in the supine position.

A previous study reported that the acetabular cup angle accuracy is better in the supine position than in the lateral decubitus position without navigation or fluoroscopy [24]. However, this finding has not been widely replicated, and its accuracy in the supine position without assistive devices remains poorly characterized. In the supine position, fluoroscopy can be performed more easily, which contributes to more accurate acetabular cup alignment [25].

Several studies have reported high acetabular cup angle accuracy using devices that do not require preoperative data, such as radiography or CT, for THA in the supine position [2, 7, 10, 15, 16, 26]. Systems that require pin insertion—such as pelvic rotation correcting device, accelerometer-based portable navigation system, three-dimensional mini-optical portable navigation, or conventional AR-based navigation system—have reported mean absolute errors ranging from 2.2° to 3.3° for inclination and 2.5° to 3.8° for anteversion [2, 7, 10, 26]. Other pinless systems, including robot-assisted systems, have also shown high accuracy, with reported mean absolute errors of 1.8° to 2.9° for inclination and 2.6° to 3.4° for anteversion [15, 16].

Although the accuracy of the novel pinless system in our study fell within these reported ranges, its primary advantages are efficiency and safety. These existing systems often lengthen operative time because of the need for pin insertion, pelvic rotation correction, or a complex registration process. Furthermore, pin-based navigation specifically is associated with a reported incidence of 0.46% for pin-site complications [12] and 1.2% for intraoperative navigation abandonment [13] in THA. Although these risks are low, avoiding them entirely would be beneficial. Therefore, the novel pinless AR-based navigation system that eliminates the risk of pin-site complications and intraoperative navigation abandonment, as well as the time burden of registration, provides a strong practical advantage.

Signed difference analysis revealed a significantly different systematic bias between the two navigation systems. The final cup inclination and anteversion angles tended to be greater in the pinless group than in the conventional group. We hypothesize that this systematic offset originates a structural limitation in registration timing. In both systems, the cup angle is measured using a smartphone attached to the cup impactor, which is reported to cause approximately 2 degrees of anterior pelvic tilt [27]. In addition, approximately 1 degree residual anterior tilt from surgical retractors is also reported even after removal [27].

While the conventional system registers the FPP before surgery and can compensate for intraoperative tilt, the pinless system defines this intraoperatively tilted position as the neutral FPP. The discrepancy between intraoperative anterior tilt and the actual neutral pelvic position on postoperative CT likely explains the larger final cup angles observed in the pinless group, as a relative posterior pelvic tilt is known to increase inclination and anteversion [28]. We hypothesize that applying an intentional correction, such as targeting a slightly lower angle, could mitigate this systematic bias when using this pinless AR-based navigation system. However, lacking clinical evidence, this remains a hypothesis for future research rather than a clinical recommendation. Interestingly, the comparable accuracy rates between the two groups suggested that this systematic offset was effectively compensated for during surgery.

Regarding the absolute differences from the target angle and the target achievement rates, the absence of a significant difference in these analyses suggests that both systems demonstrated a clinically comparable ability to achieve the target angles for both inclination and anteversion.

Robot-assisted systems and CT-based navigation have been reported to provide better accuracy for cup angle and position than portable navigation [2]. However, one report showed no significant difference in the cup angle between portable and CT-based navigation in the supine position [29]. Furthermore, whether these advanced systems improve clinical outcomes remains controversial [30–32]. Our findings were relevant to this debate. Although the novel pinless system might not have matched the higher accuracy reported by some robot-assisted or CT-based systems, the clinical outcomes in our cohort (no dislocations) suggested the view that simpler and sufficiently accurate navigation is a valuable option. Considering that robot-assisted systems and CT-based navigation are expensive [3], we believe that a simple, pinless, and portable navigation system may be a valuable option, particularly for THA in the supine position.

This study had several limitations. First, this study was a single-center, sequential nonrandomized study. Because the conventional system was used earlier in the study period and the pinless system later, this sequential design introduces the potential for chronological bias. Although all participating surgeons were highly experienced with navigation prior to the study, we cannot entirely rule out the influence of improvements in surgical workflow, increasing familiarity with the AR interface, or evolving surgical judgment over time.

Second, although postoperative CT is an accurate measurement method, variations in pelvic tilt on different examination dates could have introduced errors. To mitigate this, we excluded CT scans obtained within one month of surgery, as residual pain could affect pelvic positioning.

Third, supplemental screw fixation was performed at the discretion of the surgeon. Although previous studies have indicated that screw fixation can alter the acetabular cup angle [33], we prioritized surgical safety and efficiency by avoiding the reattachment of the impactor. Since this measurement protocol was applied consistently to both groups, we believe the relative comparison between the two navigation systems remains valid.

Fourth, although instantaneous registration is a key advantage of this pinless system, we did not record the specific time required for preparation (including pin insertion) and registration in the conventional group. However, despite the lack of time measurements, the pinless system provides a quantitative reduction in the number of surgical steps by eliminating both the invasive pin-fixation process and the need for sequential ASIS registration.

Finally, with the current computer-assisted THA techniques, the dislocation rate is extremely low [34]. In addition, we excluded the spinopelvic change cases, which are known as high risk of dislocation [35]. While we consider a follow-up period of approximately one year sufficient for a preliminary assessment given that the majority of dislocations occur within the first year [36], larger studies with longer durations are required to confirm the effectiveness of these navigation systems in reducing the risk of dislocation.

Furthermore, the aforementioned exclusion of cases with spinopelvic imbalance, along with other methodological constraints—specifically the sequential nonrandomized design, the lack of intraoperative pelvic motion measurements, and the absence of registration time data—may limit the generalizability of our findings. Therefore, future multicenter, prospective studies addressing these specific variables are warranted.

In conclusion, the pinless AR-based navigation system demonstrated acceptable cup placement accuracy; however, anteversion accuracy was slightly inferior to that of the conventional portable AR-based navigation system. While the pinless system achieved comparable target achievement rates, eliminates the risk of pin-site complications and intraoperative navigation abandonment, and offers instantaneous registration, further prospective studies are required to determine whether this difference has clinical relevance.

## Data Availability

The data presented in this study are available on request from the corresponding author due to reasonable request.
